# Effect of *Cuscuta epithymum* Acquainted with Risperidone on the Improvement of Clinical Symptoms and Cognitive Impairment in Patients with Schizophrenia: A Triple-Blind Randomized Placebo-Controlled Trial

**DOI:** 10.31661/gmj.v8i0.1334

**Published:** 2019-12-29

**Authors:** Maedeh Parvizi, Farbod Fadai, Moahammad Reza Khodaei-Ardakani, Gholamreza Amin, Leila Abdi, Mehdi Noroozi, Iman Ansari

**Affiliations:** ^1^Department of Psychiatry, University of Social Welfare and Rehabilitation Sciences, Tehran, Iran; ^2^Department of Pharmacognosy, Faculty of Pharmacy, Tehran University of Medical Sciences, Tehran, Iran; ^3^Department of Traditional Medicine, Faculty of Pharmacy, Tehran University of Medical Sciences, Tehran, Iran; ^4^Department of Psychiatry, University of Social Welfare and Rehabilitation Sciences, Tehran, Iran; ^5^Medical Students Research Committee, Shahed University, Tehran, Iran

**Keywords:** Schizophrenia, Cuscuta, Risperidone, Cognitive Impairment

## Abstract

**Background:**

*Cuscuta epithymum* (CE) is an established medicinal herb utilized for treating psychosis in Persian medicine. The aim of this study was to investigate the effect of CE combined with risperidone on the clinical symptoms and the cognitive impairment in patients diagnosed with schizophrenia.

**Materials and Methods:**

In this triple-blind randomized placebo-controlled trial, the intervention group received a dose of 500 mg of CE in the form of a capsule to be taken twice a day accompanied by an appropriate dose of risperidone. The control group was presented with a placebo identical to that of the CE capsule plus the allocated dose of risperidone. The PANSS and SCoRS questionnaires were used to assess the status of subjects prior to the initiation of the intervention as well as being put to use at the end of the second, fourth, and eighth week post-intervention. Registering and recording intel concerning positive and negative symptoms felt by participants (PANNS), and a test to assess the cognitive impairment of the individuals.

**Results:**

After eight weeks of treatment, all negative and positive symptoms besides hostility and somatic concern exhibited a significant improvement in the CE group (P <0.05). In contrast, the CE placebo group displayed no substantial improvement in the cases of the positive, negative and general symptoms (P>0.05) regarding cognitive impairment, after eight weeks of treatment, all symptoms were greatly improved in the CE group (P<0.05), while the effect of the placebo on the patients cognitive impairment remained mostly stationary (P>0.05). Consequently, after eight weeks after the intervention, we can determine that the CE treatment has been noticeably more effective at improving positive, negative and cognitive symptoms of patients with schizophrenia.

**Conclusion:**

The results of this study demonstrated that CE, possessing possible antioxidant and neuroprotective properties, safely improved the positive and negative symptoms, and cognitive impairment of patients with schizophrenia.

## Introduction

Schizophrenia is a psychiatric disorder that is characterized by the dysfunction in thought and emotion. This disorder usually presents itself in the form of hallucinations, delusions, disorganized behaviors and incoherent speech patterns, which ultimately lead to a significant disruption in social functioning [1-3]. A combination of biological, psychological, and social factors contributes to the development of schizophrenia [1]. To date, no definitive treatment for schizophrenia has been reported, and antipsychotic drugs are only used to moderate symptoms and progression of the disease; however, many of which have several side effects such as akathisia, tardive dyskinesia, acute parkinsonism, etc. that must be weighed against their benefits. Several studies on other drugs with fewer side effects are underway [4, 5]. Studies have shown that oxidative stress is associated with the pathophysiology of many psychiatric disorders, including schizophrenia. In addition, lipid peroxidation plays a role on the development of positive and negative symptoms in patients with schizophrenia [6]. Therefore, it is hypothesized that a drug with anti-oxidative properties can be effective in the treatment of schizophrenia. So, medicinal plants rich in antioxidant compounds are inclined to have fewer side effects and appear to be a better alternative choice, or at the very least feasible as complementary treatments [7, 8]. The Arabic term for *Cuscuta epithymum* (CE) is *Aftimoon* or *Shakutha* and in English it’s known by lesser dodder. CE is a member of the Cuscutaceae plant family. Persian medicine (PM) employed the use of CE as geriatric and purgative drug, primarily treating disorders associated with melancholic humor. It also had used in treating joint pain, defection in the urinary tract and gastrointestinal dysfunction. Additionally, there have been claims in its functionality to treat the nervous system as well as being a plausible contender for treating psychosis [9].

In addition, it has anti-microbial, cytotoxic, anti-convulsant, anti-urease, and hepatoprotective effects, as well as having antioxidant qualities that have been demonstrated in modern medicine, properties like possessing alkaloids, saponins, tannins, triterpenoids flavonoids etc., which may be useful in the treatment of diseases that overlap with oxidative stress [10]. Results of epidemiological studies have shown the prevalence of schizophrenia has almost doubled over the past 30 years. Moreover, schizophrenia is responsible for 13.4 million years of life lived with disability or the burden of disease globally [11, 12]. Hence any form of alleviation in the form of minimizing cost and maximizing the effectiveness of treatment is in high demand and highly necessary. Despite the recommendations of PM, based on the knowledge of current researchers no clinical trial has been conducted to examine the effectiveness of CE on schizophrenia. Therefore, the aim of this clinical trial was to investigate the plausibility of CE combined with risperidone on the scale of improvement on clinical symptoms and cognitive impairment in patients with schizophrenia.

## Materials and Methods

### Study Design and Patients

This study was a two-armed randomized triple-blind clinical trial (Registration No. IRCT20180502039508N1 URL:http://en.irct.ir/trial/30931) was conducted from February 2018 to April 2018 at Razi Psychiatric Hospital, University of Social Welfare and Rehabilitation Sciences, Tehran, Iran. Patients with schizophrenia that had been diagnosed with the use of the Diagnostic and Statistical Manual of Mental Disorders (5th edition; DSM-5) were treated with risperidone during this period [13]. Exclusion criteria were employed when individuals possessed any types of mood and neurological disorders, examples of which are as follows: bipolar disorder, depression, Parkinson’s disease, mental retardation, blindness and deafness, substance abuse 6 months before the start of the study, and of course patients who did not agree to participate in the study. It is important to note that the occurrence of any unwanted and/or major side effects led to the immediate discontinuation from the study, after the examination, diagnosis, and affirmation of two psychiatrists.

The investigators complied with the ethical guideline of the Declaration of Helsinki and the study protocol was approved by the University of Social Welfare and Rehabilitation Sciences: Ethics Committee requirements (reference number: IR.USWR.REC.1397.009). The objectives and the investigated effects and side-effects of the treatment were explained to the subjects, and informed consent was obtained from all participants and if required formal permission from their legal guardian. Participants were assured about the confidentiality of their information and that the outcome of the research would be published anonymously. In addition to this, things concerning treatment costs would be totally paid for by the researchers.

### Preparation of Drugs

The CE plant was received in the form of capsules from the Herbarium of Tehran University of Medical Sciences under the registry Code 6754 and were filled with a 500mg dose. In parallel, placebo capsules were filled up with an inert substance and their corresponding capsule was made to look identical. The capsules were packed in an indistinguishable container with a code number made to resemble the official codes provided by the Herbarium., in order to eliminate any conscious or subconscious bias or tampering by every official (ie. physician, researcher, and statistician) that came into contact with the packs were ensured to be unaware of the identity of its contents. At the end of the intervention, the significance of each code was revealed, determining whether the patient received the medication or placebo.

### Intervention

**Figure 1:**
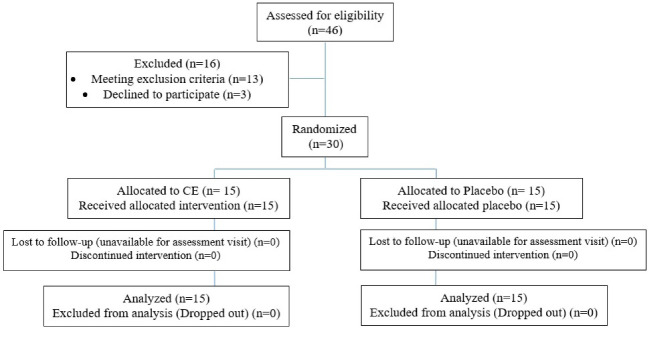
Study flowchart

After careful consideration, determining whether subjects fitted the eligible criteria, 30 admitted patients with schizophrenia were divided into two equal groups named intervention and control (with block randomization method (Figure-1). Patients were assorted to match their dosage and type current therapy with risperidone. In the intervention group, the 500 mg of CE capsule was administrated daily, and the placebo capsule was given to the control group, which was visually similar to the CE capsule. The patients were requested to advise investigators about any side effects or complaints during the study. Possible side effects were checked and registered at the baseline and each visit.

### Outcome

The information-gathering tool included a three-part checklist. The first part contains demographic information includes age, gender, and marital status. The second part contains the Positive & Negative Symptom Scale (PANSS) questionnaire. PANSS questionnaire was used to assess the positive and negative symptoms of patients based on semi-structured clinical interviews. Of the 30 items included in the PANSS, 7 constitute a Positive Scale, 7 a Negative Scale, and the remaining 16 a General Psychopathology Scale. Each of the 30 items is accompanied by a specific definition as well as detailed anchoring criteria for all seven rating points. These seven points represent increasing levels of psychopathology, as follows: “1: absent” to “7: extreme”. The validity and reliability of the Persian version of the PANSS have been confirmed previously (r=0.99) [14, 15]. The third part contains the Schizophrenia Cognition Rating Scale (SCoRS) questionnaire. SCoRS is a 20 item interview-based clinical assessment that evaluates cognitive deficits and the degree to which these deficits impair patients’ day-to-day functioning. SCoRS assesses the cognitive processes that are commonly impaired in patients with schizophrenia including memory, working memory, attention, reasoning and problem solving, language and motor skills [16]. The Persian version of this questionnaire has been reviewed and validated in Iran by Mazaheri *et al*. in 2017 on patients with schizophrenia [17]. PANSS and SCoRS were used for all patients before the intervention (giving the CE or placebo), then at the end of the second, fourth, and finally the eighth week. Clinical interviews and questionnaires were completed by the assistant.

### Statistical Analysis

SPSS Software version 18 was used to conduct statistical analysis (IBM, Chicago, IL, USA). Results were presented as mean ± standard deviation (SD). The Chi-square test was used for a statistical comparison of nominal variables in the baseline of the two groups. Paired t-test or non-parametric Wilcoxon test was used in the statistical population retrieval to determine the distribution of samples. In order to compare the two groups at baseline and at the outcome the end of the trial, independent t-test was applied. A P-value less than 0.05 was considered significant.

## Results

**Figure 2:**
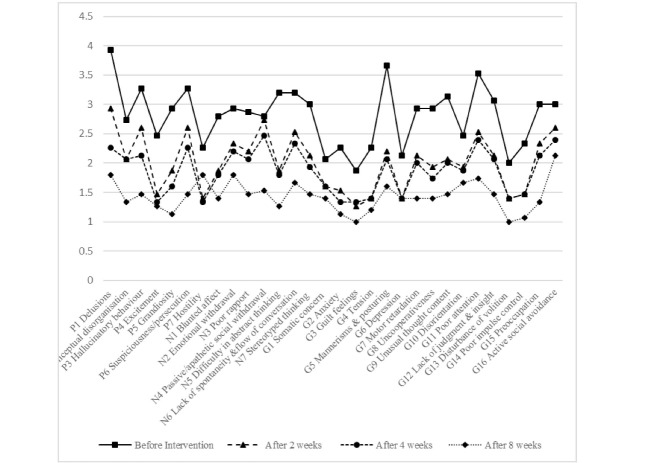
The effect of CE+risperidone on the mean amount of each parameter of positive and negative symptoms of patients based on PANSS questionnaire in different weeks compared to the pre-intervention status of patients

**Figure 3:**
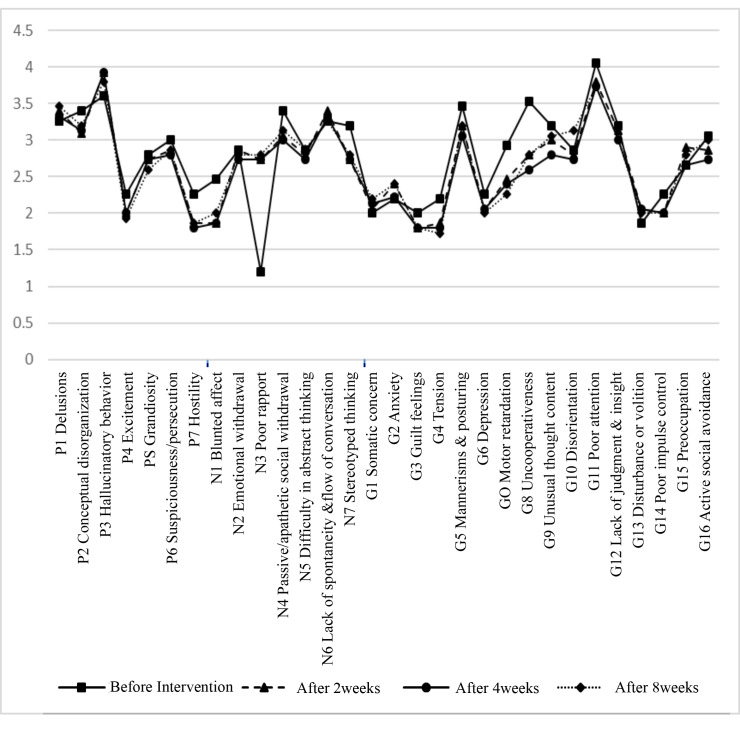
The effect of placebo+risperidone on the mean amount of each parameter of positive and negative symptoms of patients based on PANSS questionnaire in different weeks compared to the pre-intervention status of patients

**Figure 4:**
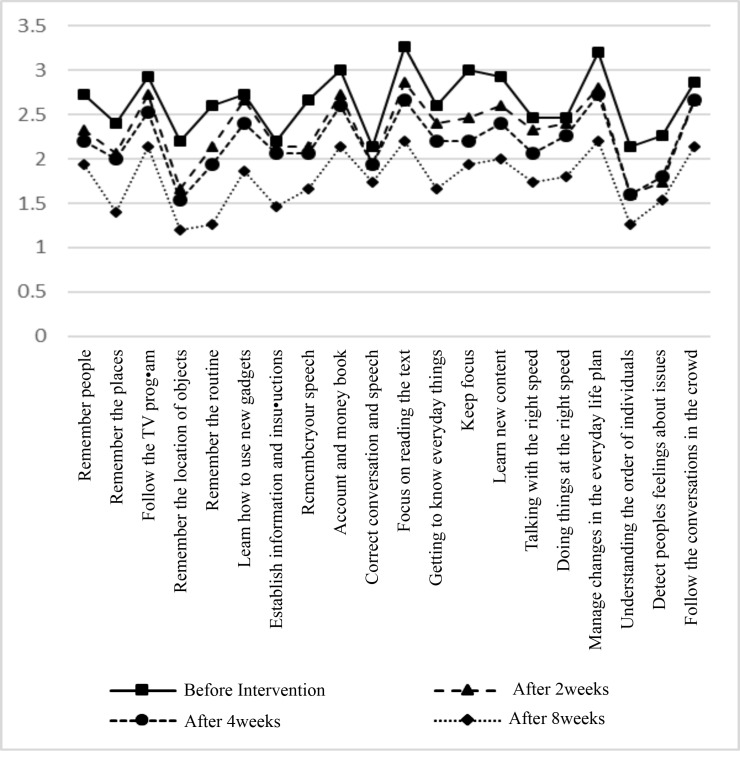
The effect of CE+risperidone on the mean amount of each parameter of cognitive status of patients based on SCoRS questionnaire in different weeks compared to the pre-intervention status of patients

**Figure 5:**
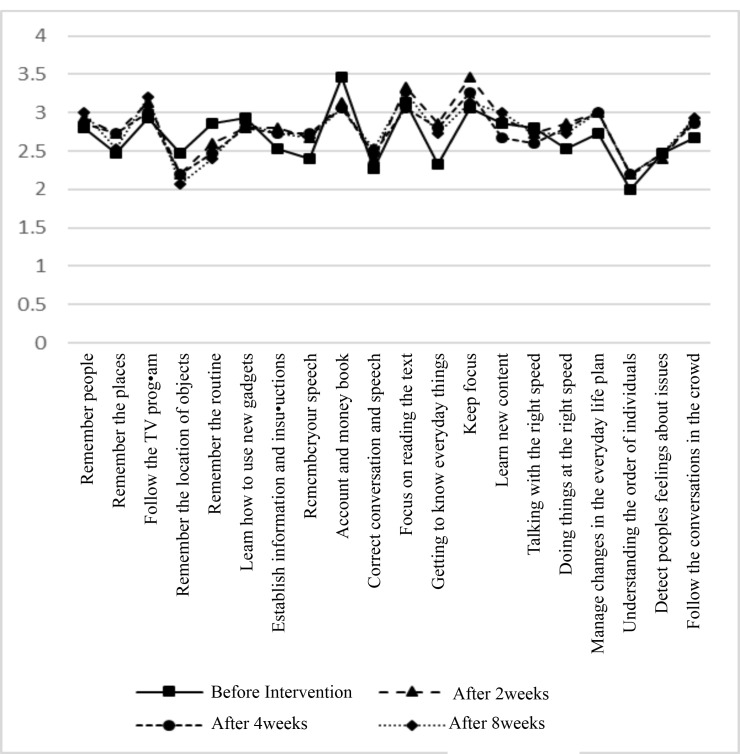
The effect of placebo+risperidone on the mean amount of each parameter of cognitive status of patients based on SCoRS questionnaire in different weeks compared to the pre-intervention status of patients

The mean age of the patients was 50.16 ± 10.92 years. Fourteen patients (46.7%) were male and 16 patients (53.3%) were female. Also, 23 patients (76.7%) were single and 7 patients (23.3%) were married. There were no statistically significant differences in terms of age, gender and marital status between two groups (P=0.33, P = 0.35, P=0.5, respectively). After eight weeks of treatment, all negative and positive symptoms except hostility and somatic concern were significantly improved in CE group (P <0.05, Figure-2). Compared to CE, placebo showed no significant improvement in most cases of the positive, negative and general symptoms (P>0.05, Figure-3). Regarding cognitive status, after eight weeks of treatment, all symptoms were significantly improved in CE group (P <0.05, Figure-4). Compared to CE, the effect of placebo on the cognitive impairment of patients was insignificant in most cases (P>0.05, Figure-5). As shown in Table-1, in the 8th week, treatment with CE compared to placebo could significantly and positively improve the positive and negative symptoms of patients with schizophrenia. In addition, Table-2 shows the comparison of the effects of CE and placebo in treatment weeks on cognitive impairment in patients. As it is shown, at the 8th week, the treatment with CE compared to placebo has been able to significantly and effectively improve the cognitive impairment of patients with schizophrenia. In addition, no serious side effect has been shown in the whole of this intervention.

## Discussion

In recent years, extensive studies have been done on the medicinal properties of different species of herbs on schizophrenia. Research shows that the combination of herbal medicines and anti-psychotics can have beneficial effects [18]. Despite the recommendations of PM to use CE in the treatment of psychosis, studies on this plant, especially regarding its antipsychotic effects, are very limited. Therefore, this randomized triple-blind clinical trial study was performed on patients with schizophrenia for eight weeks. The results of this study showed that after eight weeks of treatment with CE, almost all of the positive and negative symptoms as wells as all symptoms of cognitive impairment were significantly improved. Also, the results of this study showed that after eight weeks of treatment, CE compared to placebo could significantly improve the positive, negative and cognitive impairment of patients with schizophrenia with no serious side effects. In the presented study, it is hypothesized that CE possibly functions with three mechanisms, including antioxidant, neuroprotective and GABA-enhancing effects that play an important role in improving the symptoms of schizophrenia suffers. The results of these studies have shown that decreased inhibition plays a role in GABAergic system, which leads to overstimulation of dopaminergic neurons, in turn this stimulates excessive dopamine release and produces schizophrenic symptoms, so GABA amino acid helps to increase the inhibitory properties of central nervous system (CNS), and any action to increase GABA can help to improve schizophrenia [19, 20]. The results of the study conducted by Forouzanfar *et al* in 2018 showed that the hydroalcoholic extract of CE has a possible effect on the GABAergic system and an increase in this neurotransmitter, decreasing the Pentylenetetrazole-induced seizure in mice [21]. In addition, Gupta *et al*. showed that the Cuscutaceae family has GABA enhancing effects since the amount of GABA increased after six weeks of treatment in the brain of mice [22]. Therefore, it can be theorized that the improvement in the symptoms of patients was due to the effect of CE on GABAergic system. Recent studies have suggested that oxidative stress caused by reactive oxygen species plays a role in the development of schizophrenia. It has been shown that high doses of antioxidants can improve metabolic abnormalities in schizophrenia. Therefore, treatment with antioxidants could be used to treat this disease [19, 20]. The Cuscutaceae family like CE, has a rich content of natural antioxidants; hence, it has the potential to prove beneficial in the treatment of neurological diseases such as cognitive impairment [23, 24]. Also, the neuroprotective effects of these plants have been attributed to their antioxidant potential [25]. According to this theory, these herbs reduce inflammation, protecting neurons against ischemic cell death and increases neuronal proliferation [23]. Meanwhile, CE with its rich flavonoid and alkaloid content [10], is influential in reducing oxidative stress and protecting the CNS. On the other hand, the result of the studies showed that treatment with antioxidant agents significantly improved the symptoms of the disease with respect to the reduction of PANSS scale scores, which is consistent with the results of the present study [26]. Therefore, it can be concluded that one of the other mechanisms of CE has an additional effect on improving symptoms of the patients, most likely being its antioxidant and neuroprotective traits. The results from this study are consistent with the data derived from PM. In PM, CE has been called “*Dava al-Jonoon*” which means the cure for psychosis. According to PM, it is effective in treating seizures, headaches, melancholia, mania, psychosis, obsession, panic, palpitation, and hiccups [9, 10]. In some historic sources remaining from old Persian scientists such as *Zayn al-Din Gorgani*, Avicenna, *Muvaffak Harawi*, *Al-Biruni* it has been mentioned that there is no better treatment than CE for melancholia [9]. In this study, as well as the recommendations of PM, CE improved the psychotic symptoms of the subjects, but one must not forget that the precise mechanism of this effect is yet to be completely understood. Given the limited collaboration of patients with schizophrenia and the difficult conditions for conducting research on these patients, the limitations of this study had a low sample size and a short-term follow up from the test subjects. Despite being highly recommended by PM, CE is yet to be appreciated by the global medical community as there have been no previous studies to verify CE’s ability to positively influence psychotic diseases, thus one of the selling points of this study is that it is the first of its kind as it has targeted the plausibility of CE.

## Conclusion

The results of this study showed that CE, displayed possible antioxidant and neuroprotective effects as well as playing a role on increasing GABA, after eight weeks of treatment, it improved almost all of the positive and negative symptoms and cognitive impairment of patients with Schizophrenia. Compared with CE, the effect of placebo on cognitive impairment and positive and negative symptoms in most cases was not significant.

## Conflict of Interest

The authors of this manuscript have no conflicts of interest.

**Table 1:** Comparison of Effects of CE+Risperdone and Placebo+Risperdone on the Mean±SD of Each Parameter of Positive and Negative Symptoms of Patients Based on PANSS Questionnaire in Different Weeks on the Positive and Negative Symptoms of Patients

	**Before intervention**			**After 2 weeks**			**After 4 weeks**			**After 8 weeks**		
	**CE**	**Placebo**	**P-value**	**CE**	**Placebo**	**P-value**	**CE**	**Placebo**	**P-value**	**CE**	**Placebo**	**P-value**
P1 Delusions	3.93±1.3	3.26±1.4	0.35	2.93±0.8	3.4±1.2	0.16	2.26±0.5	3.33±1.2	0.04	1.8±0.7	3.46±1.2	0.006
P2 Conceptual disorganization	2.73±0.9	3.4±1.5	0.39	2.06±0.9	3.1±1.5	0.12	2.06±0.9	3.13±1.5	0.12	1.33±0.4	3.2±1.5	0.009
P3 Hallucinatory behavior	3.26±2	3.6±1.6	0.3	2.6±1.4	3.93±1.7	0.08	2.13±1	3.93±1.7	0.06	1.46±0.6	3.8±1.7	0.008
P4 Excitement	2.46±1	2.26±0.9	0.87	1.46±0.7	2±0.9	0.27	1.33±0.6	2±0.9	0.15	1.26±0.6	1.9±0.8	0.07
P5 Grandiosity	2.93±1.3	2.8±1.5	0.83	1.86±1	2.73±1.4	0.54	1.6±0.7	2.73±1.4	0.26	1.13±0.3	2.6±1.5	0.02
P6 Suspiciousness/persecution	3.26±1.4	3±1.4	0.63	2.6±0.7	2.86±1.6	0.1	2.26±0.6	2.8±1.5	0.08	1.46±0.6	2.86±1.5	0.07
P7 Hostility	2.26±1.1	2.26±1.3	0.82	1.4±0.6	1.86±1.3	0.58	1.3±0.4	1.8±1.3	0.45	1.8±0.5	1.86±1.3	0.17
N1 Blunted affect	2.8±1.8	2.46±1.1	0.05	1.86±1.1	1.86±0.6	0.24	1.8±1	1.86±0.6	0.43	1.4±0.9	2±0.7	0.005
N2 Emotional withdrawal	2.93±1.7	2.86±1.4	0.78	2.33±1.3	2.86±1.4	0.26	2.2±1.2	2.73±1.3	0.23	1.8±1.3	2.8±1.5	0.28
N3 Poor rapport	2.86±1.6	1.2±2.73	0.5	2.2±1.2	2.73±1.2	0.49	2.06±1.1	2.73±1.2	0.24	1.46±1.3	2.8±1.3	0.004
N4 Passive/apathetic social withdrawal	2.8±1.6	3.4±1.4	0.48	2.73±1.3	3.06±1.4	0.73	2.46±1.1	3±1.4	0.05	1.53±1.3	3.1±1.5	0.008
N5 Difficulty in abstract thinking	3.2±1.8	2.86±1.3	0.7	1.86±1	2.8±1.2	0.09	1.8±0.8	2.73±1.1	0.01	1.26±0.6	2.8±1.4	0.015
N6 Lack of spontaneity &flow of conversation	3.2±2	3.26±1.6	0.59	2.53±1.3	3.4±1.5	0.62	2.3±1.1	3.33±1.5	0.06	1.66±1.1	3.26±1.5	0.08
N7 Stereotyped thinking	3±2	3.2±1.7	0.45	2.13±1.3	2.73±1.5	0.48	1.93±1	2.73±1.5	0.4	1.46±0.8	2.8±1.4	0.056
G1 Somatic concern	2.06±1	2±0.6	0.7	1.6±1.2	2.06±1.1	0.13	1.6±1.2	2.13±1.1	0.2	1.4±1.2	2.2±1.1	0.006
G2 Anxiety	2.26±1	2.2±1	0.74	1.53±0.7	2.4±1	0.08	1.3±0.4	2.26±0.9	0.02	1.13±0.3	2.4±1.1	0.003
G3 Guilt feelings	1.86±0.8	2±0.7	0.86	1.26±0.6	1.8±0.8	0.12	1.33±0.6	1.8±0.8	0.2	1±0	1.8±0.8	0.005
G4 Tension	2.26±1.1	2.2±0.8	0.62	1.4±0.6	1.86±0.8	0.23	1.4±0.5	1.8±0.7	0.16	1.2±0.4	1.7±0.8	0.09
G5 Mannerisms & posturing	3.66±2	3.46±1.8	0.14	2.2±1.3	3.2±1.6	0.35	2.06±1.3	3.06±1.7	0.45	1.6±1.1	3.2±1.6	0.03
G6 Depression	2.13±1.4	2.26±0.8	0.14	1.4±0.6	2.06±1	0.22	1.4±0.5	2.06±0.9	0.1	1.4±0.6	2±0.9	0.25
G7 Motor retardation	2.93±1.6	2.93±1.4	0.91	2.13±1.4	2.46±1.2	0.49	2±1.1	2.4±1.18	0.57	1.4±0.6	2.26±1.2	0.17
G8 Uncooperativeness	2.93±1.7	3.53±1.5	0.46	1.93±0.9	2.8±1.6	0.54	1.7±0.8	2.6±1.5	0.45	1.4±1	2.8±1.8	0.1
G9 Unusual thought content	3.1±1.7	3.2±1.6	0.25	2.06±0.7	3±1.6	0.02	2±0.6	2.8±1.6	0.1	1.46±0.6	3.06±1.8	0.07
G10 Disorientation	2.46±1.4	2.86±1.9	0.17	1.93±0.8	2.8±1.4	0.35	1.86±0.8	2.73±1.4	0.4	1.66±0.8	3.13±1.6	0.09
G11 Poor attention	3.53±1.3	4.06±1.6	0.54	2.53±1.2	3.8±1.4	0.29	2.4±1	3.73±1.5	0.09	1.73±0.8	3.7±1.5	0.015
G12 Lack of judgment & insight	3.06±1.2	3.2±1.4	0.18	2.13±0.8	3.13±1.2	0.26	2.06±0.8	3±1.2	0.28	1.46±0.6	3±1.4	0.045
G13 Disturbance of volition	2±1	1.86±1	0.18	1.4±0.8	2.06±1.3	0.16	1.4±0.8	2.06±1.3	0.16	1±0	2±1.4	0.028
G14 Poor impulse control	2.33±1.1	2.26±1	0.83	1.46±0.6	2±1	0.25	1.46±0.6	2±1	0.25	1.06±0.2	2±1.1	0.008
G15 Preoccupation	3±1.3	2.66±1.2	0.28	2.33±0.8	2.9±1.1	0.21	2.1±0.8	2.66±1.2	0.13	1.33±0.4	2.8±1.2	0.01
G16 Active social avoidance	3±1.4	3.06±1.5	0.61	2.6±1.3	2.86±1.2	0.43	2.4±1.4	2.73±1,2	0.38	2.13±1.1	3±1.3	0.34
Total	83.1±25	85.3±23	0.64	62.1±17	78.8±26	0.3	57.3±16	78.8±26	0.3	43.6±13	80.4±27	0.56

**Table 2:** Comparison of Effects of CE+Risperdone and Placebo+Risperdone on the Mean±SD of Each Parameter of Cognitive Status of Patients Based on Scors Questionnaire in Different Weeks on the Cognitive Status of Patients

	**Before intervention**			**After 2 weeks**			**After 4 weeks**			**After 8 weeks**		
	**CE**	**Placebo**	**P-value**	**CE**	**Placebo**	**P-value**	**CE**	**Placebo**	**-P-value**	**CE**	**Placebo**	**P-value**
Remember people	2.73±0.8	2.8±1.1	0.23	2.33±1.1	2.93±1	0.42	2.2±0.9	2.86±1	0.2	1.93±0.9	3±1	0.04
Remember the places	2.4±0.8	2.46±1	0.38	2.06±0.9	2.73±0.6	0.08	2±0.9	2.73±0.6	0.05	1.4±0.6	2.53±0.6	0.001
Follow the TV program	2.93±0.8	2.93±0.8	1	2.73±0.9	3.13±1	0.6	2.53±0.9	3±1	0.48	2.13±0.9	3.2±0.9	0.03
Remember the location of objects	2.2±0.5	2.46±0.6	0.31	1.66±0.7	2.2±0.6	0.11	1.53±0.5	2.2±0.6	0.02	1.2±0.4	2.06±0.6	0.001
Remember the routine	2.6±0.9	2.86±0.5	0.33	2.13±0.9	2.6±0.6	0.18	1.93±0.8	2.46±0.7	0.16	1.26±0.6	2.4±0.6	<0.0001
Learn how to use new gadgets	2.73±0.8	4.93±0.5	0.53	2.66±0.8	2.8±1	0.66	2.4±0.7	2.8±0.8	0.31	1.86±0.7	2.86±0.8	0.02
Establish information and instructions	2.2±0.6	2.53±0.7	0.58	2.13±0.6	2.8±0.7	0.06	2.06±0.6	2.73±0.8	0.06	1.46±0.6	2.73±0.8	0.003
Remember your speech	2.66±0.9	2.4±0.8	0.7	2.13±0.8	2.66±0.9	0.28	2.06±0.8	2.73±1	0.14	1.66±0.7	2.66±0.9	0.04
Account and money book	3±1	3.46±0.9	0.4	2.73±1.1	3.13±1	0.64	2.6±1.1	3.06±1	0.42	2.13±1.1	3.06±0.8	0.05
Correct conversation and speech	2.1±0.9	2.26±0.8	0.96	1.93±0.8	2.4±0.9	0.37	1.93±0.8	2.46±0.9	0.22	1.73±0.8	2.53±1	0.11
Focus on reading the text	3.2±0.8	3.13±1.1	0.63	2.86±0.7	0.33±0.9	0.13	2.6±0.9	3.06±0.8	0.59	2.2±1	3.26±0.7	0.02
Getting to know everyday things	2.6±0.9	2.33±0.9	0.86	2.4±0.9	2.86±0.8	0.53	2.2±0.8	2.8±0.7	0.18	1.66±0.8	2.73±0.8	0.02
Keep focus	3±0.6	3.06±0.7	0.9	2.4±0.9	3.46±0.6	0.02	2.2±0.8	3.26±0.7	0.01	1.93±0.7	3.13±0.8	0.002
Learn new content	2.93±0.8	2.86±0.5	0.49	2.6±0.6	2.93±0.8	0.19	2.4±0.6	2.66±0.8	0.2	2±0.6	3±0.8	0.008
Talking with the right speed	2.46±0.9	2.8±1.2	0.02	2.33±1.2	2.73±0.9	0.16	2.06±0.9	2.6±0.9	0.26	1.73±0.7	2.73±0.8	0.03
Doing things at the right speed	2.46±0.9	2.53±0.9	0.98	2.4±0.5	2.86±0.8	0.06	2.26±0.6	2.8±0.7	0.1	1.8±0.6	2.73±0.8	0.03
Manage changes in the everyday life plan	3.2±0.6	2.73±0.7	0.25	2.8±0.6	3±0.7	0.25	2.73±0.8	3±0.7	0.52	2.2±0.7	3±0.7	0.06
Understanding the order of individuals	2.13±0.7	2±0.8	0.47	1.6±0.6	2.2±0.9	0.18	1.6±0.6	2.2±0.7	0.07	1.26±0.4	2.2±0.7	0.004
Detect people’s feelings about issues	2.26±0.9	2.46±0.7	0.5	1.73±0.7	2.4±0.8	0.16	1.8±0.7	2.46±0.7	0.12	1.53±0.6	2.46±1	0.04
Follow the conversations in the crowd	2.86±0.7	2.66±1.1	0.26	2.66±0.9	2.93±0.8	0.32	2.66±0.9	2.86±0.6	0.53	2.13±1	2.93±0.8	0.07
Total	52.6±10	52.4±11	0.58	46.8±12.6	55.3±13.7	0.4	43.6±12.3	54.1±13	0.43	34.8±11	53.4±13.1	0.33
General assessment	5.66±1.9	5.46±1.9	0.74	4.86±2.2	6±2.17	0.15	4±2.1	5.8±2.1	0.22	3±2.2	5.6±2.2	0.07
General change of individual problems	-	-	-	4.73±0.8	3.46±1.3	0.06	4.73±1	4.13±0.7	0.39	5±0.9	4±0.9	0.08
